# Supporting Youth Vaping Cessation With the Crush the Crave Smartphone App: Protocol for a Randomized Controlled Trial

**DOI:** 10.2196/42956

**Published:** 2023-01-27

**Authors:** Sherald Sanchez, Alicia Deck, Neill Bruce Baskerville, Michael Chaiton

**Affiliations:** 1 Institute of Medical Science Temerty Faculty of Medicine University of Toronto Toronto, ON Canada; 2 Ontario Tobacco Research Unit Dalla Lana School of Public Health University of Toronto Toronto, ON Canada; 3 Department of Biochemistry & Biomedical Sciences McMaster University Hamilton, ON Canada; 4 Canadian Institutes of Health Research Ottawa, ON Canada; 5 Institute for Mental Health Policy Research Centre for Addiction and Mental Health Toronto, ON Canada

**Keywords:** e-cigarettes, vaping cessation, youth and young adult health, adult, youth, effectiveness, smartphone, application, vaping, cessation, assessment, intervention

## Abstract

**Background:**

The use of e-cigarettes, or vaping, has increased exponentially in the past decade, particularly among youth. Emerging evidence indicates growing nicotine dependence among youth, revealing historically higher rates of dependence among current e-cigarette users compared to rates seen in earlier research. Despite the urgent need for youth vaping cessation interventions, there is limited knowledge about the process of vaping cessation, and few evidence-based interventions are available to young people seeking support. A notable literature review on vaping cessation resources for young people recommended technology-based interventions, such as smartphone apps and SMS text messaging services, as a promising area of vaping cessation research and intervention development.

**Objective:**

The primary aim of our study is to determine the effectiveness of the *Crush the Crave* app in supporting vaping cessation among youth recruited to the intervention arm via comparison with an assessment-only control group. The primary hypothesis is that participants in the intervention group—those using *Crush the Crave*—will be more likely to be abstinent at the 6-month follow-up point than participants in the assessment-only control arm.

**Methods:**

A 2-arm, single-blind, parallel randomized controlled trial will be conducted over 12 months. Study invitations will be sent to 600 youth (age: 16-18 years) and young adult (age: 19-29 years) e-cigarette users and randomized between an intervention arm, which will be using *Crush the Crave* (n=300), and an assessment-only control arm (n=300) in a 1:1 ratio. The primary hypothesis is that participants in the intervention group—those using *Crush the Crave*—will be more likely to be abstinent at the 6-month follow-up point.

**Results:**

Study recruitment began on March 4, 2022. Recruitment is anticipated to be completed in December 2022.

**Conclusions:**

This protocol describes one of the first-ever randomized controlled trial studies to evaluate the effectiveness of an app-based intervention for supporting vaping cessation among youth aged 16 to 18 years and young adults aged 19 to 29 years. The findings from our trial will help increase our understanding of the process of vaping cessation among youth and provide evidence on the effectiveness of an app-based intervention in helping young people quit vaping. The trial results will also have implications in the development of current and future approaches to youth vaping cessation.

**Trial Registration:**

OSF Registries osf.io/hmd87; https://doi.org/10.17605/OSF.IO/HMD87

**International Registered Report Identifier (IRRID):**

DERR1-10.2196/42956

## Introduction

The use of e-cigarettes, or vaping, has increased exponentially in the past decade, particularly among young people [1,2]. Emerging evidence indicates growing nicotine dependence among youth, revealing historically higher rates of dependence among current e-cigarette users compared to rates seen in earlier research [3-5]. Several studies have documented the growing need for vaping cessation support among youth [4-10]. In 2019, young people who did not smoke accounted for a greater number of regular vapers (as opposed to *ever-vapers*) when compared to those who smoked [11]. The COVID-19 pandemic may have exacerbated these conditions, with research suggesting an increase in substance use, including vaping, among young people [9,12,13].

Despite the urgent need for youth vaping cessation interventions, there is limited knowledge about the process of vaping cessation, and few evidence-based interventions are available to young people seeking support [6,14]. To date, much of the clinical and public health guidance on vaping cessation is adapted from the literature on smoking cessation [6,8,14]. However, while there are several commonalities between smoking and vaping, there are important differences that have received relatively little attention. A narrative review published in 2021 identified the following three key differences between vaping and smoking dependence: (1) greater variability in vaping products than in cigarettes, (2) the discreetness and convenience of vaping, and (3) greater social acceptability of vaping among youth [6,8,14]. Knowledge of these differences can inform the development of vaping cessation interventions that are effective and acceptable among young people.

Previous work in intervention research and development identified technology-based interventions as a promising area for promoting youth vaping cessation [6]. Smartphone technology and mobile software apps are highly accessible, easily scalable, and cost-effective platforms for mental health interventions [15]. Globally, young people born after the year 2000 are the top smartphone users [16,17]. Consequentially, vaping prevalence is also greatest among those aged 18 to 24 years and 15 to 17 years [11,18,19]. In fact, the use of apps for addressing mental health and substance use among youth is well documented in the literature [20-23], with apps for vaping cessation being among the latest additions [24]. However, there is currently no empirically tested smartphone app that specifically targets youth vaping cessation [24].

**Figure 1:**
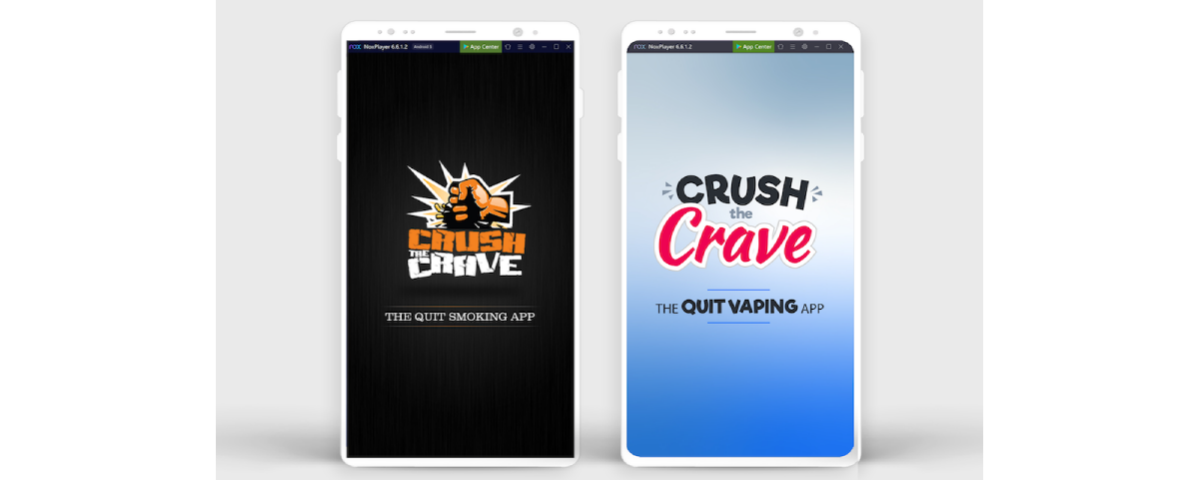
*Crush the Crave* app: comparison of the old version for young adult smoking cessation and the current version for youth vaping cessation.

This protocol describes one of the first-ever randomized controlled trial (RCT) studies to evaluate the effectiveness of an app-based intervention for supporting vaping cessation among youth. The first-ever RCT of a vaping cessation intervention was designed to evaluate the effectiveness of an SMS text messaging program called *This is Quitting* in helping young adults in the United States aged 18 to 24 years to stop vaping [8]. The trial results showed that an SMS text messaging program that provided interactive and tailored messages was successful in promoting vaping cessation among young adults [8]. However, this trial did not include youth aged 14 to 17 years, for whom vaping cessation is urgently needed, as participants [1,4,18]. In our study, 600 youths (aged 16-18 years) and young adults (aged 19-29 years) who vape will be recruited. The intervention is called *Crush the Crave*—a comprehensive, full-featured app for supporting youth vaping cessation (Figure 1).

*Crush the Crave* for vaping cessation was developed based on an earlier version of the app for smoking cessation [25-27]. An RCT of 1599 young adult smokers assessed *Crush the Crave* for smoking cessation and found that the 30-day point prevalence abstinence (PPA) at 6 months for the intervention condition (118/820, 14.4%) was not significantly different (odds ratio 0.82, 95% CI 0.63-1.08) from that for the control condition (132/779, 16.9%). However, the rate of abstinence was favorable when compared with the unassisted 30-day quit rates of 11.5% of young adults [28]. In addition, it was determined that 61% of the young adult smokers in the RCT were vaping. The increasing nicotine dependence and use of vapes among youth have prompted interest in developing an app for vaping cessation based on people’s experiences with *Crush the Crave* for smoking cessation. *Crush the Crave* is a tracker app that tracks the following information: the number of vape-free days and the amount of money saved since quitting vaping, as well as triggers and cravings. The app also provides the following functions: tailored supportive messages and inspirational graphic images for quitting vaping, personalized quit plans, graphic performance feedback, digital awards for achieving milestones, the reporting of vaping triggers, assistance for dealing with cravings, and links to other supportive resources that can aid in vaping cessation.

The primary aim of our study is to determine the effectiveness of the *Crush the Crave* app in supporting vaping cessation among youth recruited to the intervention arm via comparison with an assessment-only control group. The primary hypothesis is that participants in the intervention group—those using *Crush the Crave*—will be more likely to be abstinent at the 6-month follow-up point than participants in the assessment-only control arm. Our trial is currently open for recruitment. The anticipated completion date for our study is March 2024.

## Methods

### Trial Design

A 2-arm, single-blind, parallel RCT will be conducted over 12 months. Study invitations will be sent to 600 youth and young adult e-cigarette users and randomized between an intervention arm, which will be using *Crush the Crave* (n=300), and an assessment-only control arm (n=300) in a 1:1 ratio. The trial protocol is registered with the Center for Open Science (registration digital object identifier: 10.17605/OSF.IO/HMD87). The trial protocol was developed in accordance with the SPIRIT (Standard Protocol Items: Recommendations for Interventional Trials) 2013 checklist of recommended items for interventional trials [29].

### Ethics Approval

The study is being conducted by the Ontario Tobacco Research Unit. Approval for the study protocol was obtained from the University of Toronto Office of Research Ethics (protocol number: 00038410).

### Recruitment and Enrollment

Eligible participants are youth aged 16 to 29 years who live in Canada and have used nicotine e-cigarettes in the previous 30 days. Study participants will be recruited through the Vaping Dependence Cohort—an existing panel of youth enrolled in a prospective cohort study at the University of Toronto who provided consent for recontact in future studies at the Ontario Tobacco Research Unit. This panel is comprised of a broad sample of youth who self-reported current or past regular use of nicotine e-cigarettes. The use of nicotine e-cigarettes was assessed through a question that asked respondents if they had used e-cigarettes daily, in the past week, in the past month, in the past 3 months, or in the past year and if they had ever used an e-cigarette. This cohort was designed to balance recruitment by smoking status (*ever* or *never*), e-cigarette use (*ever* or *never*), and age (16-18 years or 19-25 years). Consequently, the prevalence of smoking, e-cigarette use, and younger participants was higher than what would be expected in a simple random sample of the population. An analysis controlling for the study design elements found that the within-group characteristics of the included youth were broadly consistent with expectations, which were based on a representative sample [9].

### Informed Consent

After eligibility screening, participants must provide informed consent to proceed with study enrollment. The web-based consent form contains information about the study, the *Crush the Crave* app, participant eligibility, requirements and expectations for study participation, compensation, data collection, and plans for protecting participants and their privacy (ie, data deidentification), as well as the contact information of study staff and the University of Toronto Office of Research Ethics. Those who agree to participate in the study will receive an email containing a link to download the *Crush the Crave* app (sent to the email addresses they provided).

### Compensation

Each participant in the intervention and control arms will receive compensation (CAD $10 [US $7.46] electronic gift card) upon completion of the baseline survey questionnaire and each time they complete a follow-up survey throughout the course of the study.

### Randomization

Eligible participants will be identified from the Vaping Dependence Cohort. The *rand()* function in Excel (Microsoft Corporation) allowed for simple randomization; the top 50% highest numbers identified the participants that will receive invitations to use the *Crush the Crave* app. A research assistant from the Vaping Dependence Cohort will conduct the randomization and create a variable with a blinded key for the initial data analysis, which will allow the investigators and analyst to be blinded to the group allocation up until the data analysis is complete.

### Interventions

#### Crush the Crave

**Figure 2:**
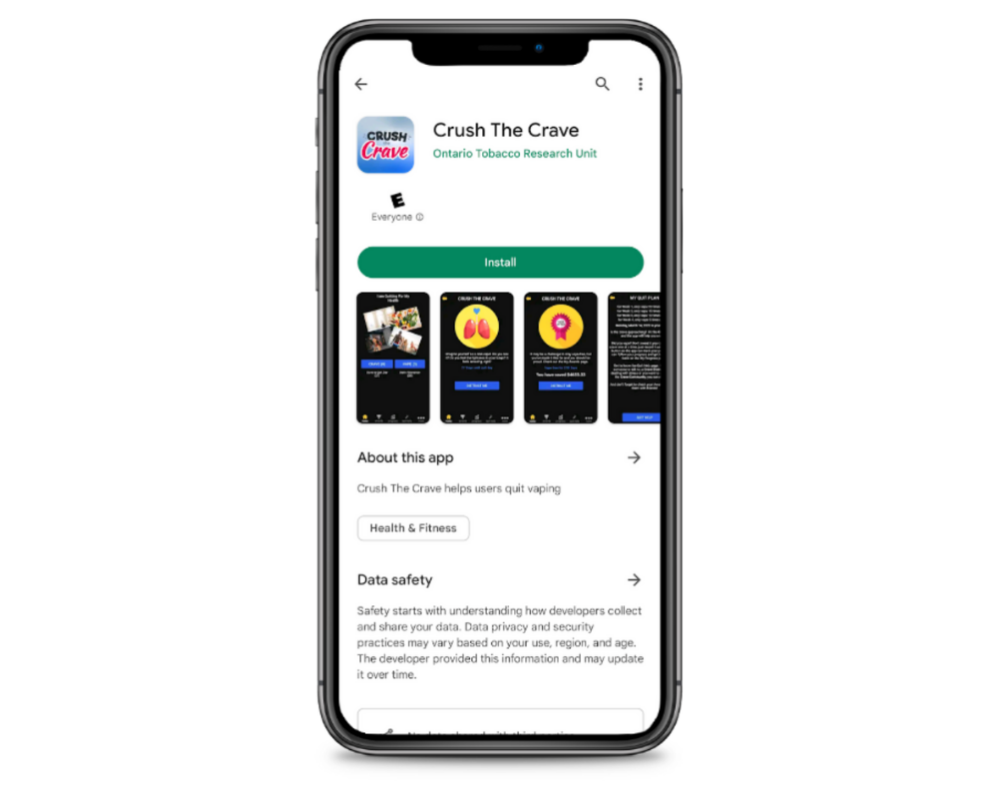
*Crush the Crave* app on the Google Play Store.

An earlier version of the *Crush the Crave* app was developed for smoking cessation [22,25], incorporating fundamentals of persuasive technology [30] and recommended practices for addressing tobacco use and dependence [31]. This evidence base formed the foundation for the current version of the *Crush the Crave* app for vaping cessation (Figure 2), which was redesigned (using principles of co-design [32]) by a multidisciplinary team of tobacco control researchers, design researchers, and software developers. Youth and young adults who vape were also engaged in the co-design process through a series of focus groups (January to February 2020) and an intensive design research workshop called *VapeHack* (May 2020).

Like the original app for young adult smoking cessation, *Crush the Crave* for vaping cessation enables users to customize a quit plan. As a tracker app, *Crush the Crave* monitors the amount of money saved and the number of vape-free days since the user’s quit date. This savings calculator is one of the most significant changes in this version of *Crush the Crave*, which involved the conversion of the money saved based on the number of cigarettes smoked in a day to the money saved based on the number vape pods used in a week (calculated as the number of vape-free days multiplied by the product of the average number of vape pods used each day and the price of vape pods in Canada). The previous version of the app had a health calculator that monitored users and informed them of the health milestones achieved since quitting smoking. In the current version of the app, the health calculator feature was removed as a reflection of the lack of definitive evidence on the health effects of vaping. Further development of *Crush the Crave* for vaping cessation will be halted during the trial.

The app also tracks cravings and vaping habits through a diary of vaping triggers that records when, where, and why vaping occurs. The app displays supportive messages and graphic images of encouragement in response to cravings and relapse, as well as direct links to evidence-based resources, such as quitlines. A visual representation of quitting progress over time is provided to users on the main page, and users have the ability to upload personalized pictures and verbal affirmations. *Crush the Crave* also gives users the ability to geotag where vaping and cravings occur, and this information is only viewable to the user. Other features include reminders or push notifications regarding the money saved, health improvements, and the achievement of goals; digital awards for reaching milestones; and supportive messages tailored to where users are in the quitting process.

#### Assessment-Only Control

Based on an approach that is similar to the one used by Graham and colleagues [8], participants will be invited via email to complete the baseline assessment, and they will be invited to complete the follow-up assessment questionnaires asking about e-cigarette use and abstinence (as outlined in the following sections) at 3, 6, 9, and 12 months. At the end of the intervention period and following the last follow-up assessment, they will be invited to try the *Crush the Crave* app, if they are interested.

### Data Collection

A baseline questionnaire will be sent to both the intervention group and the control group, using a self-administered web-based survey. This will collect comprehensive data on the history and characteristics of vaping (eg, vaping dependence, use of e-cigarettes, and self-confidence to quit), self-perceived addiction to vaping, health status (including mental health, mood, and perceived stress), and current and previous history of the use of other substances (combustible cigarettes, marijuana, alcohol, hookahs, and other tobacco products). The baseline survey will also ask questions on environmental factors and cessation goals. Self-reported demographic data (age, sex, gender and sexual orientation, province and country of residence, race and ethnicity, education, and marital status) will also be collected in the baseline survey.

A follow-up survey will be sent to the control and intervention groups at 3, 6, 9, and 12 months. Responses to the follow-up survey will be used to analyze any significant changes in vaping habits and differences between groups. The follow-up survey questionnaire includes items on cross-contamination and the use of cessation methods.

### Outcome Measures

The primary outcome variable will be self-reported 30-day PPA at 3 months, operationalized as not having vaped, even a puff, in the last 30 days. The secondary measures include the intention to quit smoking in the next 6 months (*yes* or *no*), number of puffs per vaping session, number of vape sessions per day, and number of sessions in the past 30 days.

### Process Measures

The mHealth (mobile health) App Usability Questionnaire [33] will be used to collect data on process measures from the intervention group. The mHealth App Usability Questionnaire collects data on the following three main constructs: ease of use and satisfaction (8 items), system information arrangement (6 items), and usefulness (7 items). Data from app analytics will also be used as process measures for the intervention group, including whether participants downloaded the intervention (*yes* or *no*), the frequency of use, the most frequently used features, and user reviews.

Qualitative interviews (n=25) will be conducted with participants in the intervention arm to gain insight on the usability and acceptability of the app. Interviews will be audio recorded and transcribed verbatim, and they will be analyzed in NVivo 12 (QSR International) by using a 6-step approach to thematic analysis, as proposed by Braun and Clarke [34].

### Data Analysis Plan

#### Primary Outcome

Demographic and vaping characteristics (eg, vaping dependence, use of e-cigarettes, and self-confidence to quit) will be compared between groups at baseline. The intention-to-treat principle will be followed for statistical analyses, and all participants will be analyzed in the study arm to which they were randomized. Negative binomial regression with an identity function and robust SEs will be used to test between-group comparisons of the primary outcome variable—30-day PPA at follow-up. Multiple imputation via chained equations using the observed predictors of outcomes and the predictors of loss to follow-up will be performed to impute missing outcome data to correct for any potential bias caused by missing data. The imputation model will include age, sex and gender, education, marital status, province of residence, ethnicity, vaping dependence, self-confidence to quit, perceived stress, and the intervention group. The inverse of the adjusted risk difference will provide the number needed to treat.

The sample size of 600 (inclusive of both the control arm and the intervention arm) was selected to differentiate an effect size odds ratio of 1.60, assuming a base cessation rate of 5%, an α of .05, a power of 0.8, and a 75% (450/600) response rate.

#### Secondary Outcomes

For comparisons between secondary continuous outcomes and continuous outcomes, a linear regression with an identity function will be used. The secondary analysis of missing information will use the following two approaches to handle missingness: (1) imputation using the baseline observation carried forward or the classification of nonresponders as vapers in accordance with the Russell standard [35] and (2) imputation using the last observation of vaping status carried forward for nonresponders.

#### Process Analysis

For the process measures (whether participants downloaded the intervention, the frequency of use, satisfaction, and helpfulness), a chi-square test of association will be performed for binary and categorical variables, and for the ordinal variables approximating a normal distribution, a two-tailed *t* test for independent groups will be performed.

## Results

Study recruitment began on March 4, 2022. Recruitment is anticipated to be completed in December 2022.

## Discussion

### Protocol Overview

This is the first-ever RCT study to evaluate the effectiveness of a smartphone app for supporting youth vaping cessation. The primary aim of our study is to determine the effectiveness of the *Crush the Crave* app in supporting vaping cessation among youth recruited to the intervention arm via comparison with an assessment-only control group. The primary hypothesis is that participants in the intervention group—those using *Crush the Crave*—will be more likely to be abstinent at the 6-month follow-up point than participants in the assessment-only control arm.

The *Crush the Crave* app was developed based on the best available evidence on vaping cessation and an empirical investigation on the effectiveness of an earlier version of the app for smoking cessation [22]. At the time of writing, 4 other clinical trial protocols for evaluating vaping cessation interventions among youth and young adults were registered on ClinicalTrials.gov. Of these, only 1 has been completed; the results are accessible in the published scientific literature and show that the intervention was successful (abstinence success rates of 16% for the intervention group and 8% for the control group) in encouraging young adults to stop vaping through an SMS text messaging program [8]. This study was useful in providing a benchmark for intervention effectiveness [8]. Of the 3 remaining protocols that are actively recruiting participants, 2 aim to evaluate the effectiveness of nicotine replacement therapy for vaping cessation among youth and young adults, and 1 aims to determine the effectiveness of a smartphone app called *Goal2QuitVaping*. Compared to the target sample size in our study (N=600), that of the Goal2QuitVaping study is relatively small (N=45).

### Limitations

It is important to note the potential limitations in some aspects of the design and implementation of our trial. First, most notably, our trial was designed to include an assessment-only control group. We recognize that other app-based interventions for health are typically compared against other active controls or even other app-based interventions. Our methodology was adopted from the methodology used in the only completed trial (at the time of writing) of a technology-based vaping cessation intervention [8]. Second, our study focuses on evaluating the effectiveness of the intervention among youth and young adults who vape nicotine e-cigarettes. Given the recent rise in cannabis vaping among youth, interventions that focus on cannabis vaping will need to be developed and studied rigorously. Finally, we recognize that the active intervention is publicly available on the Apple and Google app stores and that participants in both trial groups may become aware of this fact during the trial. Cross-contamination measures will be put in place to assess awareness of and engagement with the *Crush the Crave* app among control group participants.

### Conclusions

The findings from our trial will help increase our understanding of the process of vaping cessation among youth and provide evidence on the effectiveness of an app-based intervention in helping young people quit vaping. The trial results will also have implications in the development of current and future approaches to youth vaping cessation.

## Abbreviations

mHealth: mobile health
PPA: point prevalence abstinence
RCT: randomized controlled trial
SPIRIT: Standard Protocol Items: Recommendations for Interventional Trials
